# Developing a theory-based intervention to enhance physical activity in older adults with atrial fibrillation and frailty: a behavior change wheel approach

**DOI:** 10.3389/fpubh.2025.1677729

**Published:** 2025-11-28

**Authors:** Jingzhe Liu, Guangdi Xi, Dexin Chen, Jianan Xu, Meng Liu, Fuguo Yang

**Affiliations:** School of Nursing, Qingdao University, Qingdao, China

**Keywords:** AF, physical activity, moderate exercise, behavior change wheel, frailty

## Abstract

**Background:**

Physical activity is often insufficient in older adults with atrial fibrillation (AF) and frailty. Regular physical activity is crucial for these patients as it can improve AF symptom severity, enhance quality of life, delay frailty progression, and reduce the national healthcare burden. Theory-based behavior change interventions are necessary to promote physical activity. This study aimed to develop an intervention guided by the behavior change wheel (BCW) theory to improve physical activity in older patients with AF and frailty.

**Methods:**

Our study used a framework based on BCW theory to develop a behavior change intervention in eight steps. We conducted semi-structured interviews to identify the determinants of physical activity in older patients with AF and frailty, using the capability, opportunity, motivation, and behavior model and the theoretical domain framework. Appropriate intervention functions and policies addressing these determinants were selected based on the affordability, practicality, effectiveness, acceptability, safety, and equity (APEASE) criteria. Finally, suitable behavior change techniques (BCTs) were selected to translate the intervention functions into specific intervention content.

**Results:**

Our study identified 12 factors that promote and 12 factors that hinder physical activity. Based on these factors, we selected seven intervention functions and 15 BCTs, all meeting the APEASE criteria. We used a software application as the delivery mode. Finally, we developed behavioral change interventions for older patients with AF and frailty to increase physical activity levels and compliance.

**Conclusion:**

The BCW provides a systematic approach to designing behavioral change interventions that can improve physical activity, delay frailty in older patients with AF, and reduce healthcare burdens. Our findings indicate that interventions should focus on enhancing disease knowledge, teaching physical activity skills, providing social support, and using appropriate BCTs in older patients with AF and frailty. Our next step will be to conduct a feasibility study to assess the acceptability and effectiveness of these intervention programs.

## Background

Atrial fibrillation (AF) is a cardiac arrhythmia characterized by disorganized electrical activity within the atria, leading to ineffective atrial contraction and irregular ventricular contraction. As the global population ages, AF has become a significant medical and social issue worldwide (1). This growing burden challenges global health systems. AF is the most common arrhythmia in the general population (2), with the highest prevalence among older people, according to the available epidemiological data (3). An epidemiological survey in China showed that AF prevalence increases with age, reaching 5.4% in men and 4.9% in women over the age of 75 (4). The latest guidelines state that improved diagnosis, various cardiovascular risk factors, and an aging population have increased the incidence of AF (5). Since AF can lead to cerebral infarction and heart failure, older patients with AF have a significantly higher risk of stroke mortality, cardiovascular mortality, and all-cause mortality than those without AF (6). Consequently, the safety of older patients with AF is seriously threatened.

Moreover, frailty is recognized as an important predictor of cardiovascular disease in older patients with AF. Frailty is an age-related state of decreased physiological reserves, characterized by a weakened response to stressors and an increased risk of poor clinical outcomes (6). It manifests as a decrease in muscle mass and strength, reduced endurance, and a decline in the capacity of multiple organ systems (7, 8). A study indicates that the estimated prevalence of AF among older adults experiencing frailty ranges from 48 to 75% (9). Additionally, frailty has been shown to exacerbate AF (10). By 2050, AF is projected to affect over 9 million older people aged 60 and above in China (11). Therefore, managing older patients with AF and frailty is crucial.

Physical activity is one of the most important interventions for reducing the health burden of cardiovascular disease. Older patients with AF and frailty often suffer from multiple diseases, significantly impacting their physical and psychological well-being. Numerous studies and guidelines indicate that moderate-intensity physical exercise has a positive effect on the quality of life, exercise capacity, and cardiopulmonary health of these patients (12–15). Kato et al. (16) reported that moderate-intensity physical activity benefits older patients with AF and frailty due to its anti-adrenergic, anti-inflammatory, anti-ischemic, anti-arrhythmic, and antithrombotic effects, which reduce the risk of AF. Xiang et al. (17) showed that physical activity enhances exercise patients’ capacity and delays frailty. Sławuta et al. found that exercise reduces the frequency of AF symptom episodes (18). Increasing evidence indicates that exercise is a key strategy for mitigating frailty-related physical decline in older people, with the primary mechanism involving the reduction of age-related oxidative damage and chronic inflammation while enhancing mitochondrial function and actin distribution (19). Moreover, Jordy et al. noted lower engagement in physical activity among older patients with AF (20). Therefore, effective intervention strategies must be developed to enhance the physical activity of older adults patients with AF and frailty.

Currently, little is known about the factors that may promote or hinder participation in moderate physical activity among older patients with AF and frailty. Most interventions are not tailored to address these factors, resulting in low participation and adherence (21, 22). Understanding these facilitators or barriers will help healthcare professionals identify patients who are unlikely to achieve the recommended physical activity levels and develop tailored interventions. Therefore, identifying facilitators and barriers to participation in moderate-intensity physical activity in these patients is crucial for developing effective intervention strategies.

**Figure 1:**
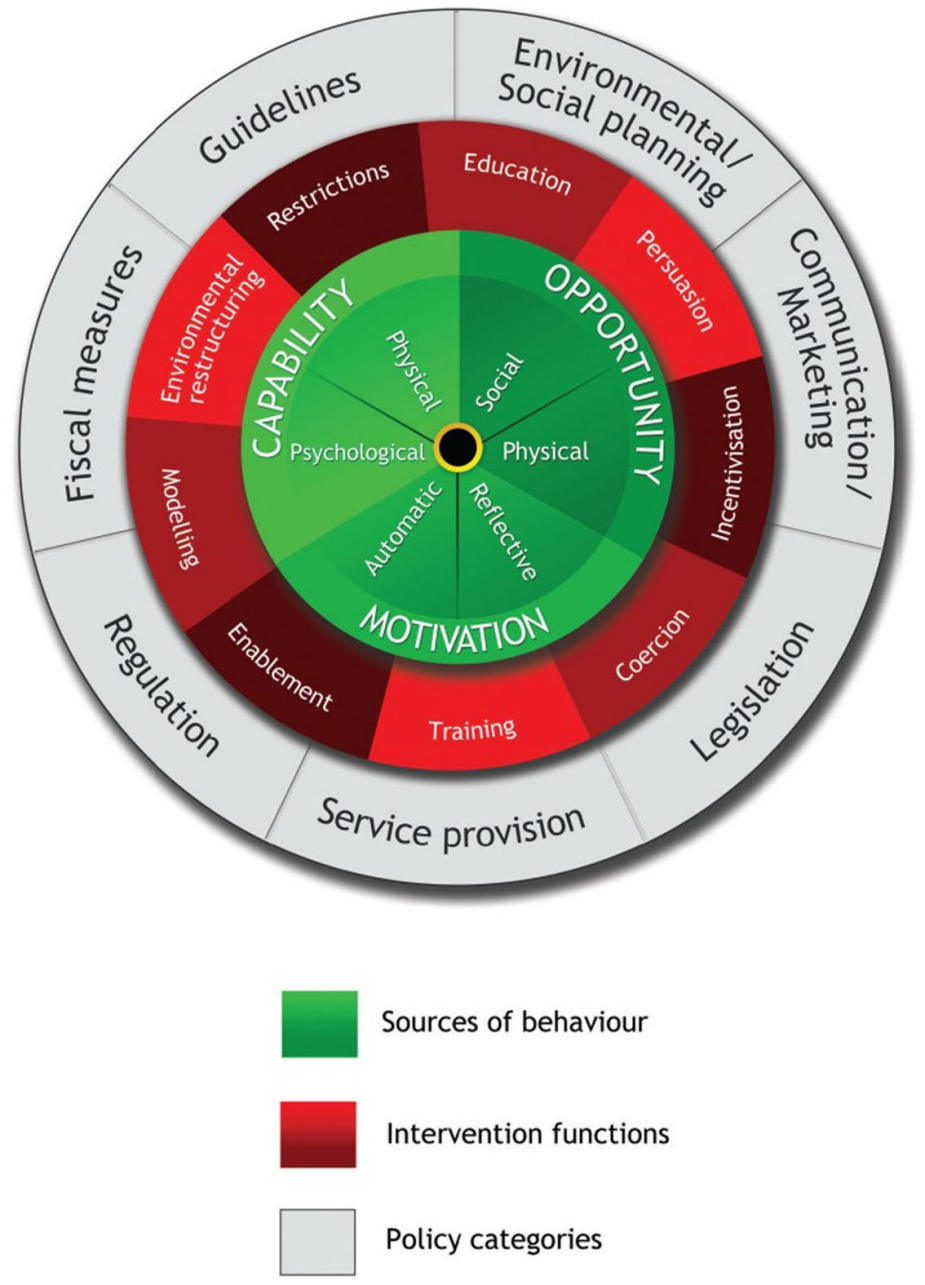
Stages of intervention development using the BCW framework (28) (adapted with permission from the authors (28), licensed under CC BY 2.0).

Previous studies have demonstrated that the behavior change wheel (BCW) theory identifies facilitators and barriers to and promotes behavior change (23–27). The BCW integrates 19 behavior change frameworks (28). It features a three-layered wheel structure (Figure 1), with the core being the capability, opportunity, motivation, and behavior (COM-B) model, which has capability, opportunity, and motivation as its basic conditions (28). The middle layer includes nine intervention functions: education, persuasion, motivation, coercion, training, restriction, environmental remodeling, modeling, and empowerment, which facilitate behavior change through interventions (28). The outermost layer encompasses seven policy categories that support intervention function implementation (28). Additionally, behavior change techniques (BCTs) are closely linked with BCW and aid in intervention design (28, 29). The theoretical domain framework (TDF) extends the COM-B model with 14 domains to further promote behavioral changes (30). However, no known study in China has applied the BCW framework to develop physical activity behaviors in older patients with AF and frailty.

According to the BCW guidelines (28, 31), constructing a behavior change intervention program using BCW theory involves three phases and a total of eight steps. Accordingly, we developed physical activity interventions using these three stages and eight steps to promote physical activity in older patients with AF and frailty.

## Methods

**Figure 2:**
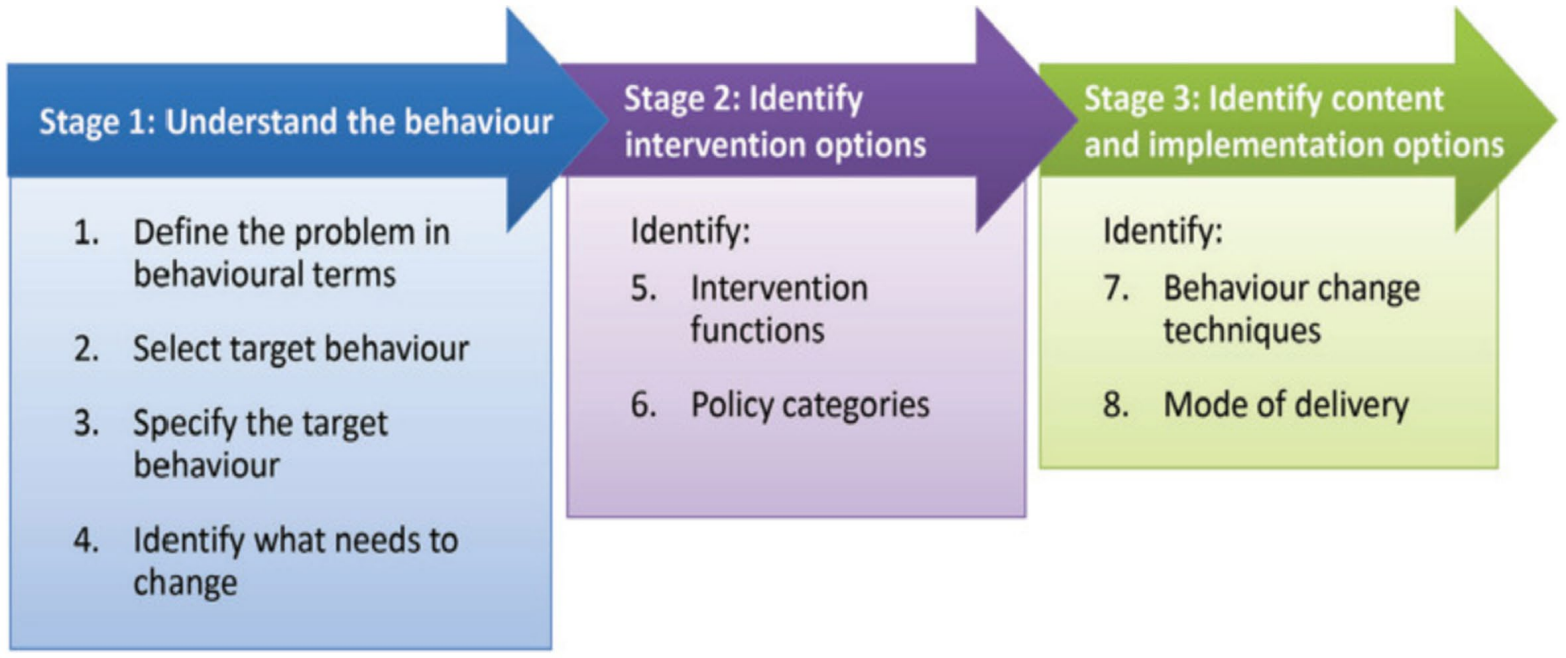
The BCW framework (adapted with permission from the authors (28), licensed under CC BY 2.0).

The BCW framework outlines an eight-step (Figure 2) process for intervention design that incorporates behavioral analysis using the COM-B model to understand and explore behavior (31). Therefore, we developed an intervention comprising three phases and eight steps.

### Stage 1: understanding the behavior

#### Step 1: define the problem in behavioral terms

Step 1 involves defining the problem using behavioral terminology to address two key questions: (1) What is the behavior? and (2) What is involved in performing the behavior? (31). Our team identified issues among older adults with AF and frailty through a literature review and discussions. We searched Cochrane Library, Embase, Web of Science, PubMed, CINAHL, Chinese National Knowledge Infrastructure, Weepu and Wanfang database for papers published through September 2023 using the following keywords: “AF,” “frailty,” “weakness,” “older patients with AF and frailty,” “physical activity,” “exercise,” “physical therapy,” “strength training,” “aerobic training,” “resistance training,” “weight training,” and “lifestyle.” Additionally, we reviewed references cited in relevant papers. The search language for keywords is exclusively in English.

#### Step 2: select target behavior

Step 2 involves selecting the target behavior aimed at addressing the issue identified in Step 1. These behaviors aim to assist older patients with AF and frailty in engaging in physical activities and achieving positive changes in physical activity behaviors. To identify these behaviors, we conducted a literature review on physical activity among older patients with AF and frailty.

#### Step 3: specify target behavior

In Step 3, we address the following questions guided by the BCW theory (31): Who should perform these actions? What needs to be done to achieve the desired changes? When to take these actions? Where to take these actions? How frequently should they occur? With whom should they be performed? In this step, we identified the target behaviors through a literature review on physical activity interventions in older patients with AF and frailty.

#### Step 4: what needs to change?

To determine the positive and negative factors of physical activity in older adults with AF and frailty, we conducted structured interviews through qualitative research. We then mapped these factors to the COM-B model and TDF to analyze what changes are needed in terms of capability, opportunity, and motivation to achieve the target behavior. The COM-B model hypothesized that the interaction of its components—capability (C), opportunity (O), and motivation (M)—drives behavior (B) (30). The TDF encompasses 14 domains derived from 33 behavioral change theories (30). By integrating the COM-B model with TDF, we can comprehensively and theoretically analyze the factors influencing behavior.

#### Participants and settings

We recruited patients diagnosed with AF and frailty aged over 60 years (diagnosed by general practitioners). Individuals with mental illness or communication difficulties were excluded. Participants were recruited through advertisements between September and December 2023. Each participant provided written informed consent. The sample size was determined based on recommendations from Francis et al. (32), with recruitment continuing until thematic saturation was reached. The advertising and recruitment were conducted at a general university hospital in Qingdao, China.

#### Data collection

Between September and December 2023, we used purposive sampling to recruit participants, aiming for diverse demographic information such as education level, income, and occupation. General participant information was gathered from the clinical information system of the hospital and semi-structured interviews were conducted individually with the participants in the hospital. We developed interview outlines using the COM-B model and TDF (Table 1), and participants consented to audio recording. The interview transcripts were coded and analyzed by the team members (XGD and XJN). Data collection continued until thematic saturation was achieved (32). Additionally, five additional participants were interviewed to ensure saturation of themes.

**Table 1:** Interview schedule.

COM-B	TDF	Question
Psychological capability	Knowledge	Do you know about AF and frailty and related physical activity?
	Behavior regulation	How do you insist on physical activity?
	Memory, attention and decision process	How do you decide to be physically active
Physical capability	Skills	What skills do you think are important for physical activity?
Social opportunity	Social influences	How do your parents, friends, or others feel about you being physically active?
Physical opportunity	Environmental context and resources	What family environment factors support or hinder you perform physical activity?
Reflective motivation	Social/professional role and identity	To what extent do you see perform physical activity as part of your role?
	Belief about capabilities	How confident do you feel about doing physical activity?
	Beliefs about consequences	What do you think you’ll get out of doing physical exercise?
	Optimism	Do you think performing physical activities will get you better?
	Intention	Do you have an idea of physical activity? (prompt: if not, why not?)
	Goals	What goals will you have when you perform a physical activity?
Automatic motivation	Reinforcement	What motivates you to do physical activity?
	Emotion	Do you think that consistent physical activity gives you a special feeling or emotion?

COM-B: capacity, opportunity, motivation-behavior; TDF: theoretical domain framework; AF: Atrial fibrillation.

#### Data analysis

We analyzed the contents of the interviews using multiple themes and a thematic analysis approach (33). Initially, XGD and XJN thoroughly reviewed and comprehended the collected clinical data and interviews to generate codes and generalize themes. The themes and subthemes were refined and defined through group discussions. Subsequently, we mapped these themes to the COM-B model and TDF. Discrepancies that emerged during this process were resolved through further discussion.

### Stage 2: identifying intervention options

#### Step 5: identifying intervention functions

This step involved identifying suitable intervention functions from the BCW framework. BCW outlines nine primary intervention functions, including education (increasing knowledge or understanding), persuasion (using communication to evoke positive or negative emotions or stimulate action), training (imparting skills), restriction (using rules to reduce opportunities for engaging in target behavior), incentivization (creating an expectation of reward), coercion (creating an expectation of punishment or cost), environmental restructuring (changing the physical or social context), enablement (increasing means/reducing barriers to enhance capability or opportunity), and modeling (providing an example to aspire or emulate) (28, 31). We identified factors that hinder and promote physical activity in older patients with AF and frailty by analyzing the COM-B model and TDF. Based on these factors, we selected appropriate intervention functions aligned with the BCW theoretical framework and screened using the affordability, practicality, effectiveness, acceptability, safety, and equity (APEASE) criteria (31). Expert consultation informed our selection process, and any disagreements were resolved through discussions. Intervention functions that did not meet the APEASE criteria were excluded.

#### Step 6: identifying policy categories

Step 6 involved considering which policy categories would support the delivery of the identified intervention functions (28, 31). BCW outlined the policy categories as “communication/marketing,” “guidelines,” “fiscal,” “regulation,” “legislation,” “environmental/social planning,” and “service provision.” However, since changing policies was not the primary focus of this study, we did not delve deeply into this step.

### Stage 3: identifying content and implementation options

#### Step7: identifying BCTs

BCW offers specific BCTs to facilitate behavioral changes (29). BCT taxonomy version 1 encompasses 93 diverse BCTs (29). We selected BCTs suitable for older patients with AF and frailty through group discussions and the APEASE criteria (29, 31). These BCTs are observable and replicable (29).

#### Step8: model of delivery

Step 8 involved identifying the delivery method (31). Here, we selected an appropriate intervention delivery method for the participants. We considered various options, including face-to-face interaction recordings, applications, phone calls, and text messages. Following group discussion and expert consultation, we chose a delivery model appropriate for the participants and met the APEASE criteria (31).

### Expert consultation

We designed the intervention functions and corresponding interventions based on the BCW framework (31). Subsequently, we conducted an expert consultation. We sent emails to experts who met our criteria through online searches and academic databases, including senior nursing staff, behavioral science specialists, general practitioners, rehabilitation therapists, and university professors. We distributed all intervention details to 15 experts and received their feedback through email within two weeks. Based on their comments, we made the necessary revisions to the manuscript.

### Ethical consideration

This study was approved by the Medical Ethics Committee of the Affiliated Hospital of Qingdao University (grant ID: QDU-HEC-2023224) and adhered to the principles of the Declaration of Helsinki. Informed consent was obtained from all the participants.

## Results

### Step 1: define the problem in behavioral terms

We identified 154 pieces of literature through the search. After screening 144 research papers were excluded and the remaining 10 papers were included. Our review of five studies indicates that physical activity is crucial in treating various cardiovascular diseases and that moderate regular physical exercise considerably reduces the risk of AF (12, 16, 34–36). Additionally, three meta-analyses underscore the necessity of personalized exercise recommendations tailored to different older patients with AF (37–39). These findings indicate that moderate exercise not only forms an essential part of non-drug treatment for patients with AF but can also prevent new cases of the conditin. Furthermore, both resistance and aerobic endurance exercises are effective strategies for preventing and treating frailty, benefiting older adults by improving physical capability (18, 40). Based on these insights, our study defines the primary challenge a developing a physical activity program specifically for older patients with AF and frailty.

### Step 2: select target behavior

Several studies have provided recommendations on physical activity types for older patients with AF and frailty (41–44). For instance, one study recommended that patients with frail should engage in at least 30 min of moderate-intensity aerobic and resistance exercises for at least 4 to 5 d per week (45). Similarly, a Korean study indicates that patients with AF may achieve optimal cardiovascular benefits with a weekly exercise totaling 1,000–1,499 metabolic equivalents of task (MET), a level of energy expenditure equivalent to 170–240 min of moderate-intensity exercise per week, but no benefit was observed when the weekly exercise total was > 1,500 MET (39). Both guidelines recommend that older patients with AF and frailty perform at least 150 min of moderate exercise per week or meet the recommendations for moderate-intensity physical activity (12, 15). Elliott et al. (46) reported that moderate-intensity physical activity reduces the risk of adverse events in patients with AF. Therefore, we selected the following target behavior and habit formation: 30 min of moderate physical activity (15 min aerobic and 15 min anaerobic) at least 5 d per week for 16 weeks.

### Step 3: specify the target behavior

We identified the target behaviors using the six questions listed in Table 2. These six questions were based on guidance from Michie et al. and group discussions (31).

**Table 2:** Identify target behavior.

What target behavior?	Promote physical activity in older adults patients with AF and frailty
Who needs to perform the behavior?	Older adults patients with AF and frailty
When will they do it?	When convenient to the older adults patients with AF and frailty
Where will they do it?	At home and hospital
How often will they do it?	30 min of moderate-intensity physical activity minimum of 5 days a week for 16 weeks
With whom will they do it?	Individual or group

### Step 4: what needs to change?

We used the COM-B model and TDF to conduct behavioral diagnoses in older patients with AF and frailty (Table 3). Through interviews with 20 older patients with AF and frailty, we identified 24 themes, including facilitators and barriers (Table 3). The patient demographics are presented in Table 4. Among them, the identified areas needing change include lack of knowledge about AF, lack of knowledge about physical activity, lack of self-control, the perception that AF is severe, absence of physical activity skills, lack of supportive physical activity environment, effects of weather, the perception that physical exercise is not important, lack of confidence, intention without action, no goals for physical activity, and negative emotions affecting physical activity.

**Table 3:** Behavioral analysis based on the TDF and the COM-B model.

COM-B	TDF	Theme	Quote	Negative Or positive
Psychological capability	Knowledge	Lack of knowledge of AF	I only know that the heart has a problem, but do not know what kind of disease.	Negative
		Lack knowledge about physical activity	I do not know what kind of physical activity to do. I also do not know how long to do physical activity.	Negative
	Behavior regulation	Lacking self-control	I’m not in the habit of physical activity.	Negative
	Memory, attention and decision process	Hobby driven	I sometimes like to walk in the park.	Positive
		Emotional driven	I’m willing to do something when I’m in a good mood.	Positive
		Self-perception that atrial fibrillation is severe	I do not like to do activities when I’m not feeling well.	Negative
Physical capability	Skills	Lack of physical activity skills	I do not know what kind of physical activity is good for me.	Negative
Social opportunity	Social influences	Social support	I want my family to support me in being physically active and remind me when I forget.	Positive
			I wish I could take a walk with my friends sometimes.	Positive
Physical opportunity	Environmental context and resources	Lacking physical activity atmosphere	I feel a little bored doing physical activities by myself.	Negative
		The influence of weather	I do not really want to go out in high temperature and rainy days.	Negative
		Sufficient time	I have so much time in retirement that I do not know what to do every day.	Positive
Reflective motivation	Social/professional role and identity	Physical exercise is not important	I think there are many things more important than physical exercise.	Negative
	Belief about capabilities	Sufficient confidence	I can do exercise every day. I’m just worried about physical activity affecting the disease.	Negative
	Beliefs about consequences	Good spirits	I feel very happy after every exercise.	Positive
		Physical fitness is improved	My physical fitness improves after exercise.	Positive
		Be beneficial to sleep	I will fall asleep more easily after I exercise.	Positive
	Optimism			
	Intention	High intention	I keep exercising in order to have a good body.	Positive
		Have intention but lack of action	I plan to exercise but sometimes cannot perform.	Negative
	Goals	No goals	I do not have a specific plan.	Negative
		Keep healthy	I just want my body to be healthy.	Positive
Automatic motivation	Reinforcement	Hope the body health	I will feel very healthy after I exercise.	Positive
		Family and friends	They sometimes suggest me to do some physical exercises.	Positive
	Emotion	Negative emotion affects physical activity	I do not want to be physically active when I’m in a bad mood	Negative

**Table 4:** Demographics of the sample (*n* = 20).

Variable	Category	*n* (%)	Mean (SD)
Sex	Male	13 (65.0)	
	Female	7 (35.0)	
Age			68.42 ± 5.12
Residence	City	12 (60.0)	
	Countryside	8 (40.0)	
Religion	Yes	5 (25.0)	
	No	15 (75.0)	
Education level	≤Primary school education	5 (25.0)	
	Middle school education or High school education	4 (20.0)	
	technical secondary school Junior college	3 (15.0)	
	≥University education	8 (40.0)	
Pre-retirement occupation	Government or public institution	3 (15.0)	
	Staff	2 (10.0)	
	Workers	3 (15.0)	
	Freelancers	5 (25.0)	
	Housewife	2 (10.0)	
	Peasant	5 (25.0)	
Co-residents	Parents	0 (0)	
	child	4 (20.0)	
	Spouse	15 (75.0)	
	Live alone	1 (5.0)	

### Step 5: identifying intervention functions

Using the APEASE criteria (31), we selected seven out of the nine intervention functions: education, enabling, training, environmental restructuring, persuasion, modeling, and incentivization (Table 5). We implemented the content of education and training through general practitioners, university professors, and senior nursing staff. Persuasion was conducted by senior caregivers and general practitioners. Enablement and modeling provide role models for older patients with AF and frailty. Environmental restructuring creates conducive conditions for physical activity in older patients with AF and frailty. Incentivization motivates older patients with AF and frailty to engage in regular physical activity. We excluded restrictions, as we did not establish rules to enforce physical activity, and coercion, as it is unacceptable for older patients with AF and frailty.

**Table 5:** Selecting intervention functions.

Intervention function	Does intervention meet APEASE criteria?	Behavioral target
Education	Yes	Psychological capability and reflective motivation
Persuasion	Yes	Automatic and reflective motivation
Incentivisation	Yes	Automatic and reflective motivation
Coercion	No	Not acceptable
Training	Yes	Physical and psychological capability
Restriction	No	Not practical
Environmental restructuring	Yes	Physical and social opportunity, automatic motivation
Modeling	Yes	Automatic motivation
Enablement	Yes	Physical and psychological capability, physical opportunity, automatic motivation

### Step 6: identifying policy categories

Identifying relevant policy categories was beyond the scope of this study. Our primary focus was on developing the physical activity intervention itself rather than on the broader policy context. Once an intervention is proven effective, incorporating policy considerations will be crucial for broader implementation and sustainability.

### Step 7: identifying BCTs

We identified 15 BCTs, including information about health consequences (5.1), prompts/cues (7.1), self-monitoring of behavior (2.3), goal setting (behavior) (1.1), demonstration of the behavior (6.1), instruction on how to perform a behavior (4.1), feedback on the behavior (2.2), behavioral practice/rehearsal (8.1), restructuring the social environment (12.2), credible source (9.1), behavioral contract (1.8), goal setting (outcome) (1.3), action planning (1.4), reducing negative emotions (11.2), and social support (emotional) (3.3). Other BCTs were ruled out through group discussions and the APEASE criteria, with reasons including unaffordability and impracticality, as well as ineffectiveness, based on prior experience. Additionally, some BCTs were deemed unacceptable to patients.

### Step 8: model of delivery

Several studies have shown that software applications (APPs) can improve physical activity levels (47–49).

Studies found that interventions delivered through APPs increase physical activity (50–52). APP-based adjunctive interventions can also effectively improve health outcomes, such as weight management (53, 54). Additionally, these APPs are convenient, smart, and fast and provide health services anywhere and anytime. We also considered traditional intervention methods such as face-to-face, telephone, and text messaging. However, APPs offer distinct advantages over these methods, including real-time monitoring of physical activity and providing timely feedback. APPs also have the added advantage of objectively tracking physical activity levels, reducing self-reporting bias. Several researchers have used APPs to deliver physical activity interventions (55, 56). Moreover, these APPs can integrate various modules like video learning and knowledge parks to enhance intervention effectiveness. After deliberation, our research team opted to use an APP as the delivery mode.

### Expert consultation

Table 6 presents the components of the intervention using the COM-B model, TDF, barriers, intervention features, BCTs, and intervention content. The main components of the intervention delivered using the APP include: (i) providing knowledge of AF and frailty, emphasizing the benefits and necessity of physical activity; (ii) assisting patients in developing a personalized plan and setting goals for physical activity; (iii) informing patients to record the duration and type of each physical activity session; (iv) setting reminders on the APP for patients to their completed physical activities; (v) instructing patients on specific aerobic and anaerobic exercises to perform, detailing each movement; (vi) conducting weekly follow-ups with the patients to address any challenges they may encounter; and (vii) providing psychological counseling to help patients manage negative emotions. Experts’ feedback on the intervention content and format, received through email, included the following suggestions:

**Table 6:** The intervention content identified based on BCTs.

COM-B	TDF domains	Barriers	Intervention functions identified	BCT & corresponding code	Intervention content and format
Psychological capability	Knowledge	Lack of knowledge of AF and frailty	Education	Information about health consequences (5.1)	Provides knowledge about AF and frailty, physical activity through the app
		Lack of knowledge about consistent physical activity			
	Behavior regulation	Lack of self-monitoring	Education	Information about health consequences (5.1)	Provide information about the negative factors and benefits of physical activity in older adults patients with AF and frailty via the app
				Prompts/cues (7.1)	Set reminders for individuals to record the type, time and/or intensity of physical activity performed each time through the app
			Enablement	Self-monitoring of behavior (2.3)	Record the type, time, and/or intensity of each physical activity performed through the app
Physical capability	Skills	Fear of physical activity aggravating conditions	Training	Demonstration of the behavior (6.1)	Provide examples of physical activities that can be watched repeatedly via the app
		Absence of physical activity skills		Instruction on how to perform a behavior (4.1)	Provide guidance on physical activity through the app
				Feedback on the behavior (2.2)	Tell people what they are not doing well in physical activity through app
				Behavioral practice/rehearsal (8.1)	Encourage individuals to practice correct physical activity through app
Physical opportunity	Environmental context and resources	Lack of time	Environmental restructuring	Prompts/cues (7.1)	Set reminders of physical activity through the app
		Lack of atmosphere for physical activity		Goal setting (behavior) (1.1)	Help participants set plans for physical activity through the app
		Weather disturbance		Restructuring the social environment (12.2)	Suggest the user through the app to make friends with people take exercise together
				Prompts/cues (7.1)	Set reminders to start physical activity through the app
			Education	Information about health consequences (5.1)	Provide information about the negative factors and benefits of physical activity in older adults patients with AF and frailty via the app
Reflective motivation	Social/professional role and identity	Physical activity is unimportant	Education	Information about health consequences (5.1)	Provide the benefits of engaging in physical activity and the negatives of not engaging in physical activity via the app
			Persuasion	information about health consequences (5.1)	Allow health care providers to introduce the physical benefits of doing physical activity via the app
	Optimism	Not perceiving benefits of physical activity	Education	Information about health consequences (5.1)	Provide the benefits of engaging in physical activity via the app
			Persuasion	Credible source (9.1)	Presentations by healthcare professionals, using the app to highlight the benefits of physical activity for older patients with AF and frailty
	Intention	Low intention	Education	Information about health consequences (5.1)	Provides knowledge about AF and frailty, physical activity through the app
		Have intention but lack of confidence		Prompts/cues (7.1)	Set reminders to start physical activity through the app
			Persuasion	Credible source (9.1)	Presentations by healthcare professionals, using the app to highlight the benefits of physical activity for older patients with AF and frailty
			Incentivization	Feedback on behavior (2.2)	Inform the patient daily of physical activity data, such as the number of steps per day and energy expend
				Self-monitoring of behavior (2.3)	Record the type, time, and/or intensity of each physical activity performed through the app
			Modeling	Demonstration of the behavior (6.1)	Provide examples of physical activities that can be watched repeatedly via the app
				Behavioral contract (1.8)	Sign contracts with participants to ensure they engage in regular physical exercise through the app.
	Goals	No goals	Enablement	Goal setting (behavior) (1.1)	Set goal of physical activity via the app
				Goal setting (outcome) (1.3)	Set a long-term goal of physical activity through the app
				Action planning (1.4)	Make a schedule for physical activity via the app
Automatic motivation	Emotion	Negative emotion affects physical activity	Enablement	Reduce negative emotions (11.2)	Provide ways to reduce negative emotions, such as listening to music via the app
				Social support (emotional) (3.3)	Encourage participants to engage in physical activities with their family or friends through this app.

*“Videos demonstrating physical activities suitable for older patients with AF and frailty” should be added*

*I just want my body to be healthy.*

*“A feature for daily weight recoding” should be added*

*“APP needs to provide observable role models for moderate physical activity in older patients with AF and frailty” should be added.*

*“Provide patients with dietary instructions to prevent malnutrition” should be added*

*“Give the patient instructions for physical activity.” should be revised to “Instruct the patient on what type of aerobic and anaerobic exercise to perform, including details of each movement.”*

Experts suggested incorporating additional intervention methods, such as mobile phone calls and text messages alongside the APP. Based on their feedback, we have implemented these revisions.

## Discussion

This study aimed to develop a behavioral intervention to increase physical activity levels among older patients with AF and frailty in China, marking the first report on the application of BCW in this population. Our study strictly adhered to the BCW theory framework. Using the COM-B model and TDF, we identified facilitators and barriers to physical activity in older patients with AF and frailty. These findings offer valuable insights for professionals studying physical activity in this demographic. Additionally, we designed an intervention to address the lack of physical activity in older patients with AF and frailty.

In our intervention, screened using the APEASE criteria, we applied seven intervention functions and 15 BCTs to promote physical activity among older patients with AF and frailty.

Our study found a widespread lack of knowledge among participants regarding AF and frailty. This finding is consistent with a previous study indicating that patients with AF and older adults generally lacked an understanding of their conditions (57). This phenomenon may stem from an insufficient focus on these conditions among the older population. To address this issue, we implemented the intervention function of education aimed at enhancing participants’ knowledge of AF and Frailty. Furthermore, our findings revealed that participants also lacked knowledge about physical activity, leading to limited engagement in physical activity. Lack of social support from family and friends further hindered the motivation to participate in sports activities. Consistent with a recent systematic review showing that social support increases physical activity participation (58), we implemented measures to enhance social support among participants, thereby promoting greater physical activity engagement.

Our study also found that restructuring the social environment can increase adherence to physical activity. In this study, we encouraged participants to connect with friends who enjoy exercising, thereby reshaping their social environments. Additionally, we found that older adults often struggle to maintain physical activity due to memory challenges, leading us to implement APP-based reminders for daily physical activity. Furthermore, self-monitoring enhanced participants’ engagement in physical activity (59). Hence, by tracking its type, duration, intensity, and frequency, adherence to physical activity was improved.

In our interviews, some participants intended to engage in physical activity but lacked the confidence to maintain their commitment. Therefore, we enhanced participants’ confidence through the intervention functions of persuasion and modeling, which consequently encouraged physical activity. However, some participants either did not intend to engage in physical activity or lacked specific goals, perceiving it as unimportant. This lack of intention and knowledge stems from older adults’ unfamiliarity with the disease and the benefits of physical activity. Therefore, healthcare professionals should prioritize health education for older adults regarding AF and frailty, covering disease definitions, risks, benefits of physical activity, and assistance in setting activity goals. Moreover, our interviews revealed a positive mood can enhance the degree of adherence to physical activity, consistent with previous research (60).

In this study (55), six intervention functions (education, persuasion, motivation, training, environmental remodeling, and empowerment) were used to promote physical activity. Kwok et al. (61) used a modeling intervention function that significantly increase physical activity levels in older adults. Therefore, our study has seven intervention functions (education, persuasion, motivation, training, modeling, environmental reshaping, and empowerment). Additionally, we identified 15 BCTS from qualitative data. In two other studies (62, 63), researchers identified 43 and 19 BCTs, respectively, to promote behavior change. Currently, there is significant research using BCW and BCTs to enhance physical activity. Future research should focus on identifying the most effective BCTs specifically for promoting physical activity behaviors in older patients with AF and frailty using APPs.

Furthermore, the unique challenges posed by the comorbidity of AF and frailty—such as concerns about bleeding risk potentially limiting exercise intensity, and the interplay between fatigue associated with frailty and symptoms of AF—highlight the need for more targeted assessments. For instance, one study emphasizes the integration of anticoagulation management with physical activity guidance (64), a perspective not yet fully incorporated into our current intervention protocol. Meanwhile, regarding the selection of intervention strategies, although our study integrated seven intervention functions, our approach appears relatively limited in modifying the physical environment (e.g., accessibility of community facilities) and leveraging policy support compared to an intervention conducted among a hospitalized patient population in the UK (65). This comparison reveals potential directions for strengthening future intervention schemes.

### Limitations

While our qualitative study identified a subset of facilitating factors and barriers, there were some limitations. First, our participants were exclusively from Shandong Province in China, limiting generalizability to older patients with AF and frailty outside of this region. Second, we only identified a subset of facilitating factors and obstacles; hence, our findings may not encompass all relevant factors. Third, the qualitative nature of our study introduces subjectivity in data interpretation. Fourth, our application of the APEASE criteria did not involve multidisciplinary teams, potentially impacting the comprehensiveness of our assessment. Fifth, our study did not consider policy categories, highlighting the need for future studies to conduct policy analyses aimed at establishing regulations to enhance physical activity among older patients with AF and frailty. Last, the effectiveness of BCW interventions in practice may pose challenges that warrant further investigation.

### Future research

Informed by BCW theory, we identified essential components for designing interventions to enhance physical activity among older patients with AF and frailty. Next, we will engage software engineers to develop application features aligned with these intervention components. Subsequently, we plan to implement and evaluate the effectiveness of this intervention in enhancing physical activity among older patients with AF and frailty.

## Conclusion

To our knowledge, this is the first application of BCW to enhance physical activity among older patients with AF and frailty, potentially improving physical condition and quality of life. This study identified 15 BCTs aimed at addressing barriers and facilitators of physical activity. Future research should determine the effectiveness of these BCTs in increasing the duration of physical activity and achieving favorable health outcomes for older patients with AF and frailty.

## Data Availability

The raw data supporting the conclusions of this article will be made available by the authors, without undue reservation.
